# Potential objective diagnostic biomarker platform of serum proteins for major depressive disorder:a preliminary exploration

**DOI:** 10.1192/j.eurpsy.2021.871

**Published:** 2021-08-13

**Authors:** S. Chen, Y. Zhang, Y. Yuan

**Affiliations:** 1 Department Of Psychosomatics And Psychiatry, ZhongDa Hospital, School of Medicine, Southeast University, Nanjing, China; 2 School Of Nursing, Nanjing University of Chinese Medicine, Nanjing, China

**Keywords:** major depressive disorder, biomarkers, multiple serum proteins, diagnostic platform

## Abstract

**Introduction:**

Major depressive disorder (MDD) is a severe, disabling condition with unknown etiology. Misdiagnosis is common when clinical symptomology criteria are used solely. Considerable evidence suggests that the upregulation of inflammatory factors and cortisol, and a decrease in neurotrophic factors, are involved in the pathogenesis of MDD.

**Objectives:**

This study explored the application of platforms composed of these serum proteins in the objective diagnosis of MDD.

**Methods:**

Serum samples from all participants including 30 MDD patients and 30 well-matched healthy controls were collected at enrollment, eight serum proteins selected initially according to previous studies were analyzed with ELISA. A logistic regression model with these proteins was built to construct the diagnostic platform for the MDD and the receiver operating characteristic (ROC) curve was used to analyze the diagnostic potential of the model.

**Results:**

Among the eight selected proteins, three (TNF-alpha, IL-6 and IL-1beta) were removed because the measurements in more than 1/3 participants were below the detectable limits of ELISA kits. Forward logistic stepwise regression analysis screened out three serum proteins including BDNF, cortisol and IFN-gamma to build the model. The regression equation was Z = 1/[1 + e−(1.438+0.005(BDNF)-0.049(cortisol)-0.007(IFN-gamma))], and the diagnostic efficacy of thees three proteins-combined achieved an area under the ROC curve of 0.884 with sensitivity of 86.7% and specificity of 83.3%.

**Conclusions:**

The results of this study provided a more reliable method to diagnose MDD, and the combination of serum BDNF, cortisol and IFN-gamma may provide an objective diagnostic platform for MDD.

